# Persistent Asymptomatic Pancreatic Hyperenzymemia or Gullo's Syndrome: A Case Report

**DOI:** 10.7759/cureus.115846

**Published:** 2026-09-06

**Authors:** Aniruddh Patel, Gargi Patel, Namrata Patel, Ruta Patel, Nikhil Patel

**Affiliations:** 1 Gastroenterology and Hepatology, Jivandeep Hospital, Anand, IND; 2 Internal Medicine, Jivandeep Hospital, Anand, IND; 3 Gastroenterology and Hepatology, Pramukhswami Medical College, Anand, IND

**Keywords:** benign pancreatic hyperenzymemia, clinical case report, exocrine pancreas, gene mutations, gullo's syndrome

## Abstract

Elevated pancreatic enzymes usually point towards pancreatic disease. In the absence of supporting clinical and imaging findings, interpretation of such elevation is challenging. We report the case of a 53-year-old man who presented with incidentally detected asymptomatic elevation of serum amylase and lipase. He was then investigated extensively without yielding remarkable laboratory or imaging findings. He was closely followed up for two years to determine the benign course of the syndrome. He remained asymptomatic without any additional imaging findings. A diagnosis of benign pancreatic hyperenzymemia or Gullo's syndrome was made. Benign pancreatic hyperenzymemia is a syndrome characterized by persistently elevated pancreatic enzymes in the absence of any underlying pancreatic disease (amylase ranged 236-520 U/L and lipase 257-956 U/L over 28 months, and two endoscopic ultrasonography (EUS) examinations (24 months apart) showed stable non-diagnostic lobularity without honeycombing). It is a diagnosis of exclusion. Treatment is reassurance and avoidance of costly re-investigations.

## Introduction

Pancreatic enzymes are widely used to diagnose pancreatic conditions. However, abnormal results can occur with many non-pancreatic conditions as well [1]. Hence, interpreting isolated elevation of amylase and lipase in the absence of correlating clinical and imaging findings remains a challenge. Elevated pancreatic enzymes with normal imaging require additional investigations to establish or rule out early chronic pancreatitis or extra-pancreatic causes.

We present the case of a rare and benign condition leading to persistently elevated pancreatic enzymes, i.e., Gullo's syndrome. Gullo's syndrome represents an idiopathic, non-progressive, and benign pancreatic condition [2]. Incidentally detecting elevated pancreatic enzymes in an asymptomatic patient with normal pancreatic imaging characterizes the presentation of Gullo's syndrome [2-9].

## Case presentation

A 53-year-old man presented to the gastroenterology clinic in January 2024 with elevated amylase and lipase detected during a routine full-body check-up two months previously. He reported absence of abdominal pain, weight loss, steatorrhea, back pain, or any other symptoms. His medical history is significant for hypertension (on amlodipine), dyslipidemia (on aspirin and atorvastatin), and type 2 diabetes (on dapagliflozin, acarbose, metformin, glimepiride, and linagliptin). He had no significant surgical history or family history. He had no history of consumption of alcohol and tobacco in any form. His general and systemic examinations were unremarkable. The laboratory reports in the full-body check-up showed unremarkable complete blood count, serum electrolytes, liver functions, renal functions, viral markers, vitamin levels, lipids, tumor markers, urine, and stool (Table 1). His diabetes was well controlled. However, serum amylase and serum lipase were elevated. Ultrasonography of the abdomen showed grade 1 fatty liver and a normal biliopancreatic system (Figure 1). Liver elastography suggested F0 fibrosis (6.06 kPa) (Figure 2).

**Table 1:** Laboratory tests for the full-body check-up

Investigations	Patient values	Reference range
Hemoglobin	15.7 g/dL	13-17 g/dL
Total red blood cells	5.45x10^6^/microL	4.5-5.5x10^6^/microL
Total leukocyte count	7.37x10^3^/microL	4.0-10.0x10^3^/microL
Platelet count	181x10^3^/microL	150-410x10^3^/microL
HbA1c	6.1%	<5.7%
Urine ketone	Absent	Absent
Urinary bilirubin	Absent	Absent
Nitrite	Absent	Absent
Urinary red blood cells	Absent	0-5 cells/HPF
Urinary leukocytes	Absent	0-5 cells/HPF
Estimated glomerular filtration rate	92 mL/min/1/73 m^2^	≥90 mL/min/1.73 m^2^ (normal)
Serum sodium	139.2 mmol/L	136-145 mmol/L
Serum chloride	103.4 mmol/L	98-107 mmol/L
Serum calcium	9.68 mg/dL	8.8-10.6 mg/dL
Magnesium	2.15 mg/dL	1.90-3.10 mg/dL
Serum amylase	236 U/L	28-100 U/L
Serum lipase	257 U/L	5.6-51.3 U/L
Thyroid-stimulating hormone	2.1 microIU/mL	0.35-4.94 microIU/mL
Creatinine	0.96 mg/dL	0.72-1.18 mg/dL
Uric acid	4.43 mg/dL	4.2-7.3 mg/dL
Urea	28.21 mg/dL	17-43 mg/dL
High-sensitivity C-reactive protein	0.7 mg/L	<1 mg/L
Lipoprotein A	12.6 mg/dL	<30 mg/dL
Apolipoprotein A1	143 mg/dL	86-152 mg/dL
Apolipoprotein B	67 mg/dL	56-145 mg/dL
Total cholesterol	133 mg/dL	<200 mg/dL
High-density lipoprotein cholesterol	46 mg/dL	40-60 mg/dL
Low-density lipoprotein cholesterol	66 mg/dL	<100 mg/dL
Very-low-density lipoprotein cholesterol	28 mg/dL	5-40 mg/dL
Triglyceride	143 mg/dL	<150 mg/dL
Alkaline phosphatase	71.26 U/L	45-129 U/L
Bilirubin total	0.6 mg/dL	0.3-1.2 mg/dL
Aspartate aminotransferase	18 U/L	<35 U/L
Alanine transaminase	17 U/L	<45 U/L
Gamma-glutamyl transferase	34.2 U/L	<55 U/L
Albumin	4.3 gm/dL	3.2-4.8 gm/dL
Globulin	2.7 gm/dL	2.3-3.4 gm/dL
Human immunodeficiency virus	Negative	Negative
Hepatitis B surface antigen	Negative	Negative
Anti-hepatitis C virus	Negative	Negative
Carbohydrate antigen 19-9	17 U/L	<37 U/L
Carcinoembryonic antigen	0.9 ng/mL	<3 ng/mL
Prostate-specific antigen	0.5 ng/mL	<4 ng/mL
Vitamin B-12	222 pg/mL	187-883 pg/mL
Vitamin D (total)	41.8 ng/mL	30-100 ng/mL
Folate	16.5 ng/mL	>5.3 ng/mL
Stool for occult blood	Negative	Negative

**Figure 1:**
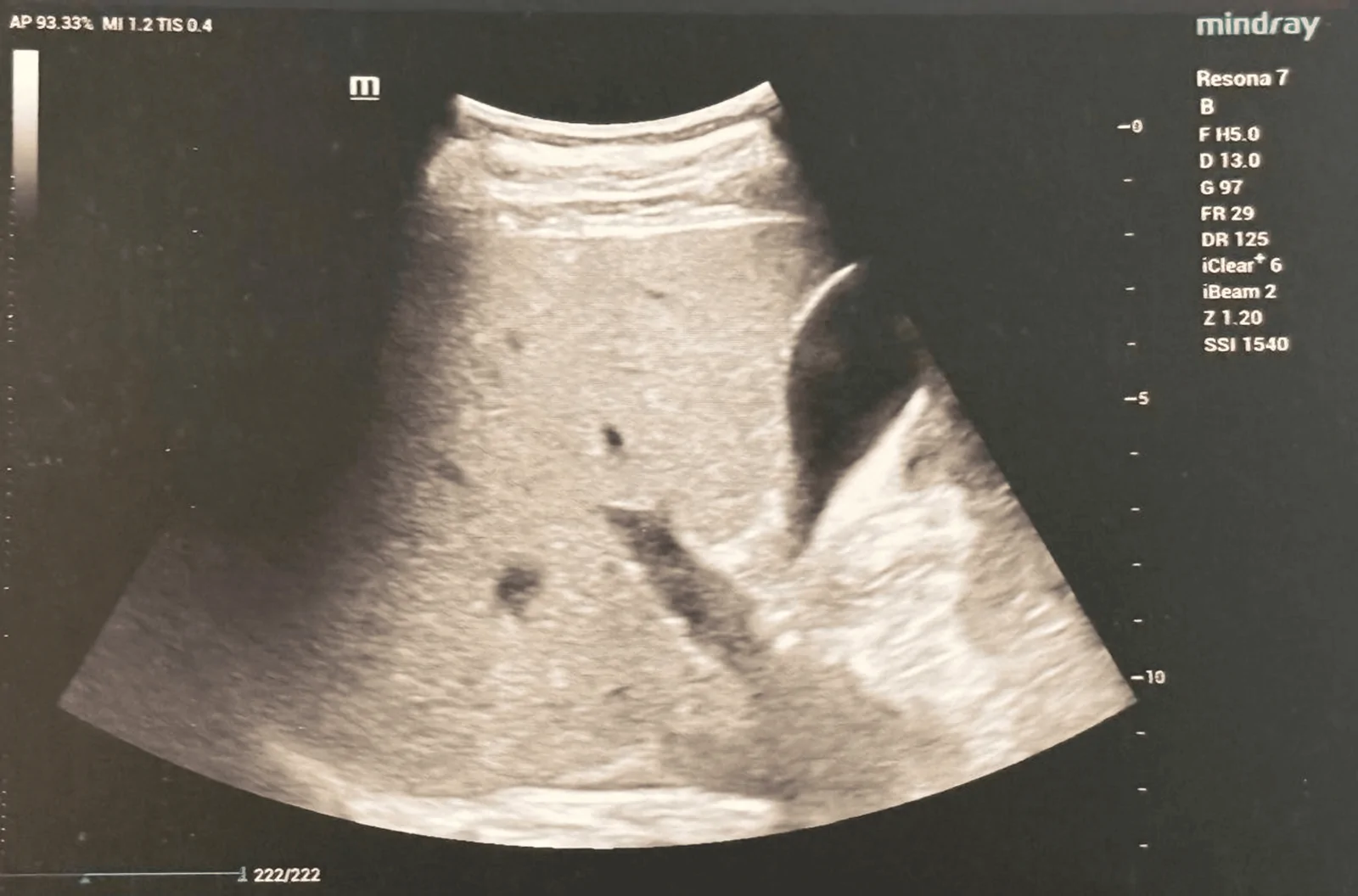
Ultrasonography of the abdomen showing grade 1 fatty liver

**Figure 2:**
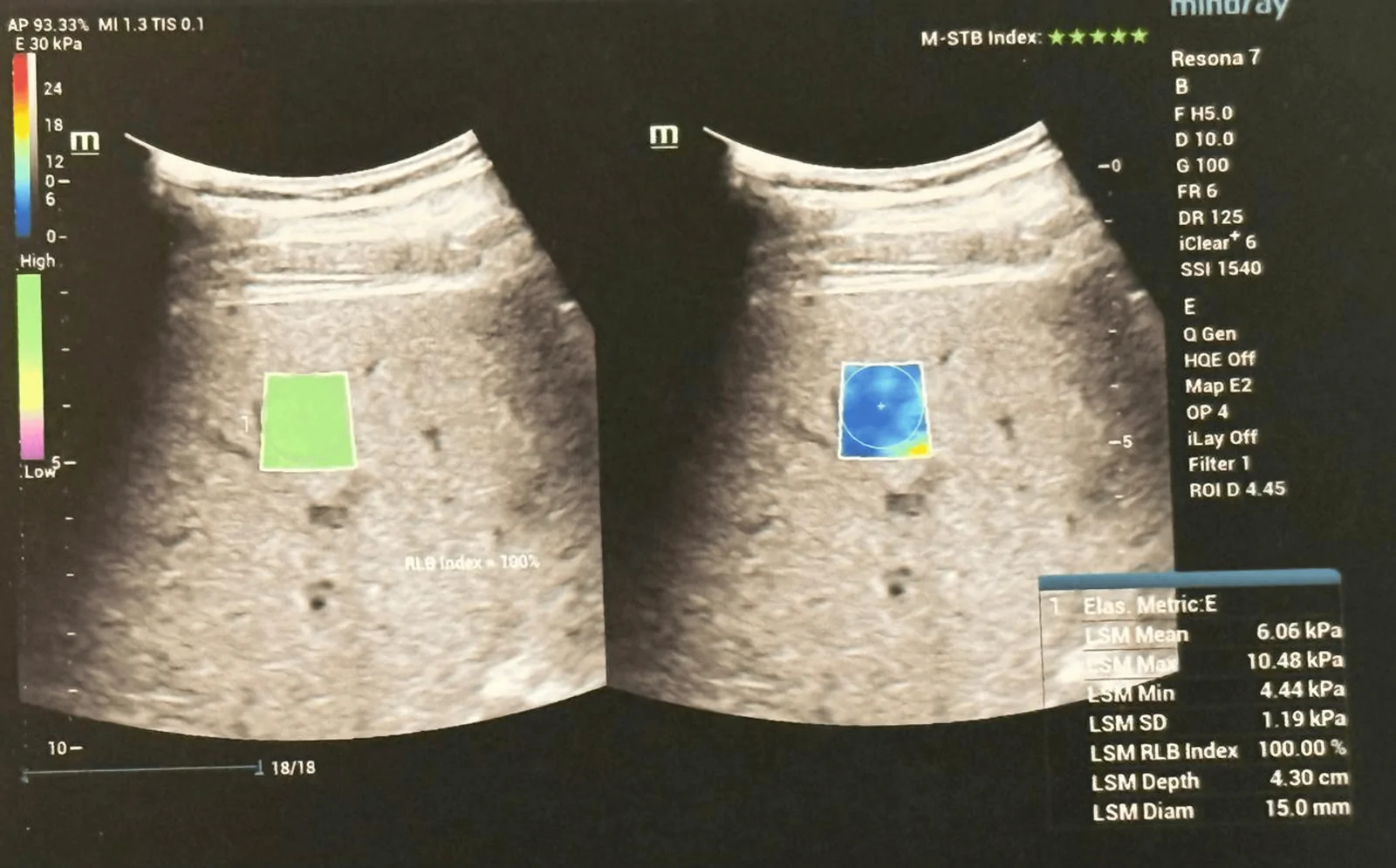
Liver elastography showing F0 fibrosis

Further imaging was done using multiple modalities to evaluate the presence of pancreatic pathology and ductal abnormalities such as Santorinicele and pancreas divisum. Contrast-enhanced computed tomography (CECT) of the abdomen (Figure 3), magnetic resonance imaging (MRI) of the abdomen (Figure 4), and magnetic resonance cholangiopancreatography (MRCP) (Figure 5) showed a normal pancreaticobiliary system and no additional abnormalities. Endoscopic ultrasonography (EUS) was done to evaluate pancreatic parenchyma for changes of early chronic pancreatitis which showed the presence of lobularity without honeycombing and no other additional features (according to the Japanese Pancreatic Society (JPS) in 2019, this is insufficient to diagnose early chronic pancreatitis) (Figure 6) [10].

**Figure 3:**
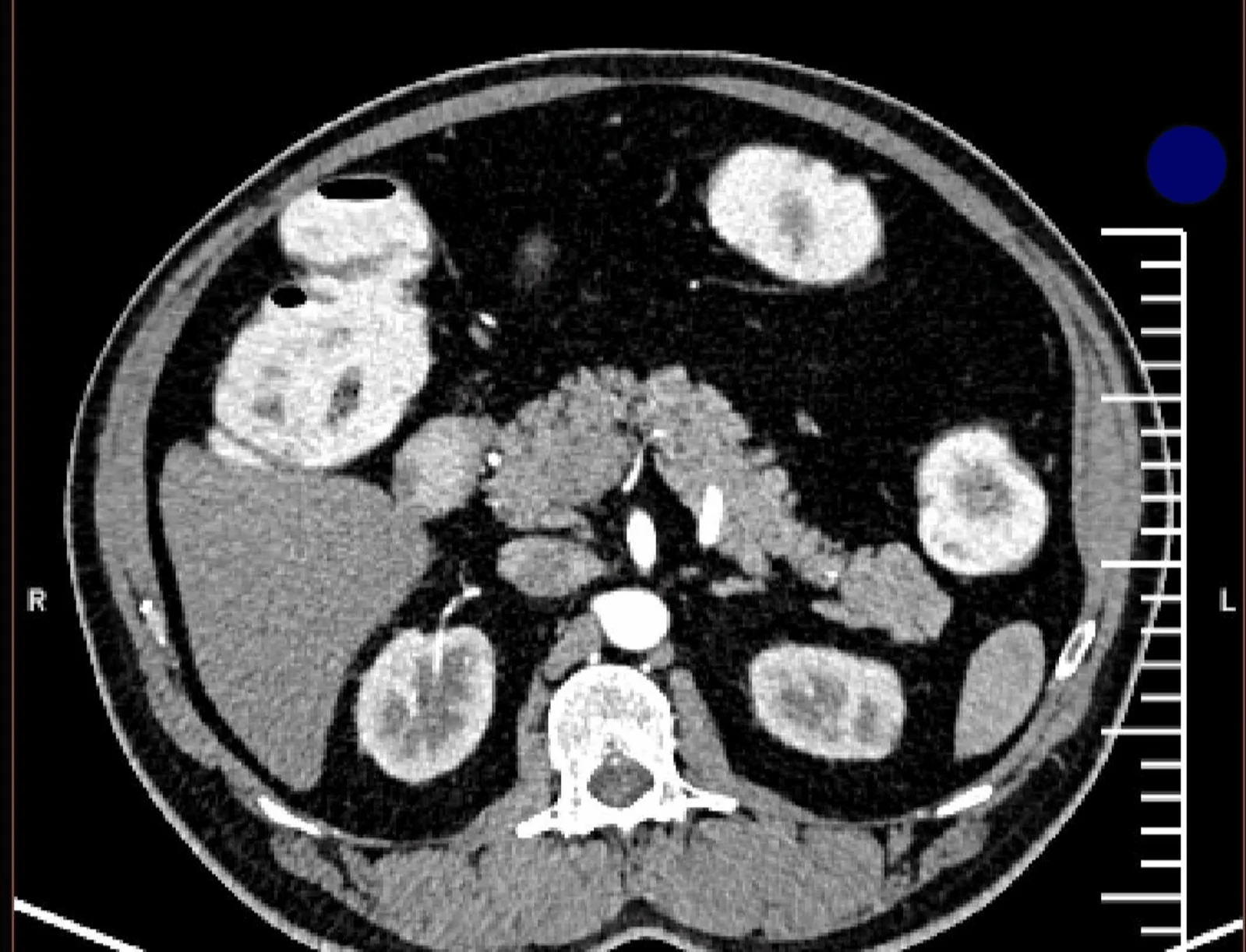
Computed tomography of the abdomen showing normal pancreas

**Figure 4:**
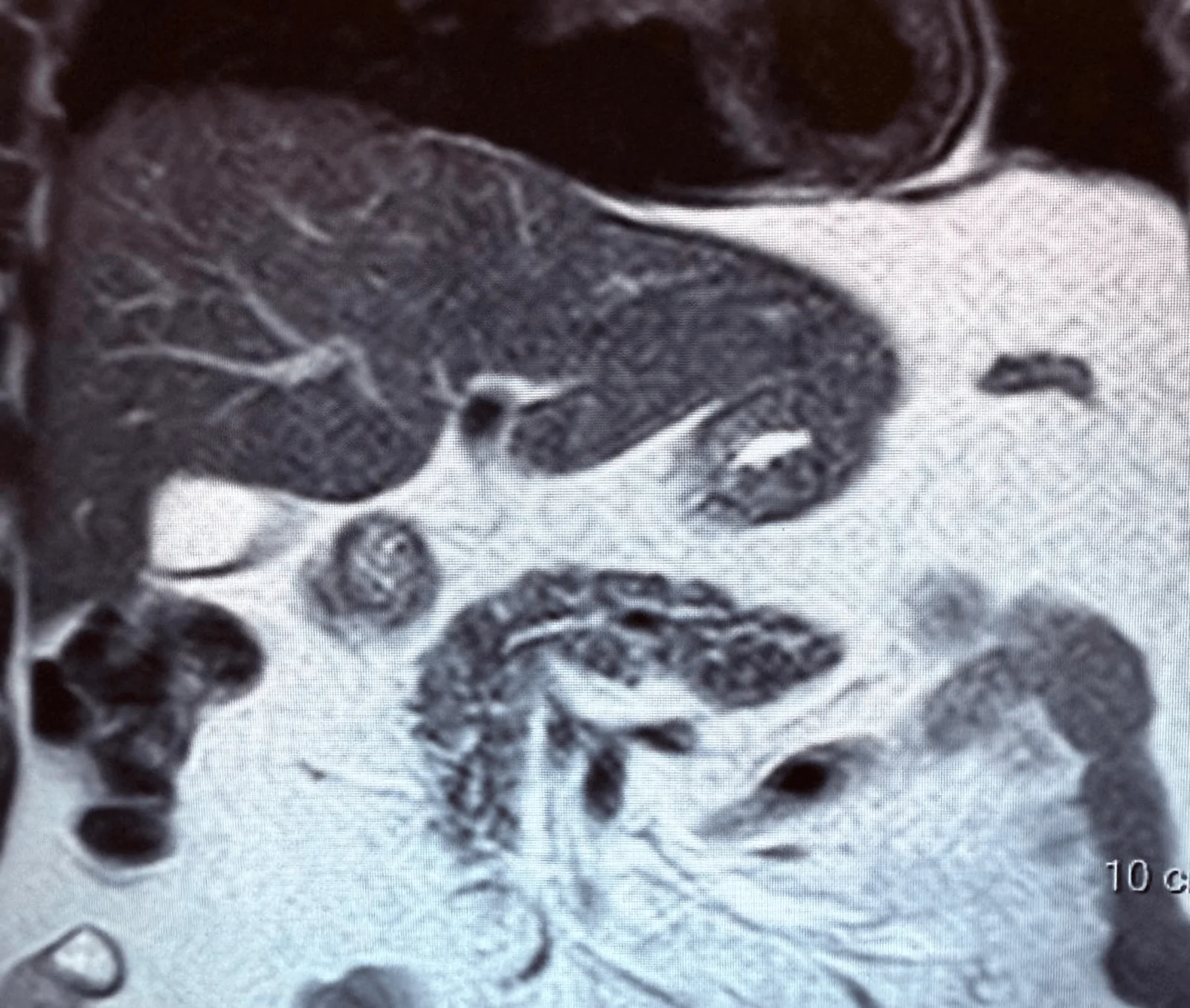
T2-weighted magnetic resonance imaging of the abdomen showing normal pancreas

**Figure 5:**
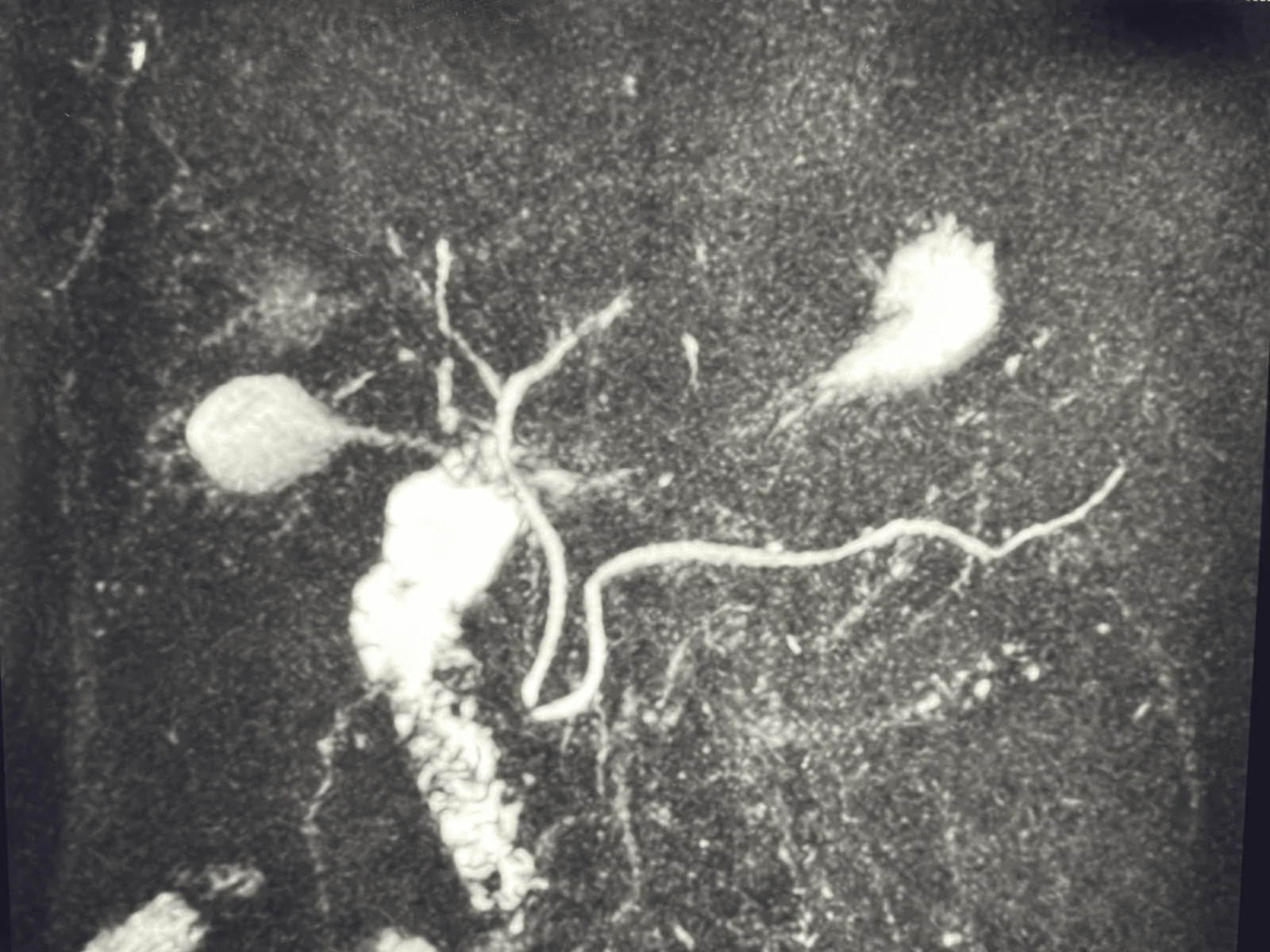
Magnetic resonance cholangiopancreatography showing normal pancreaticobiliary ducts

**Figure 6:**
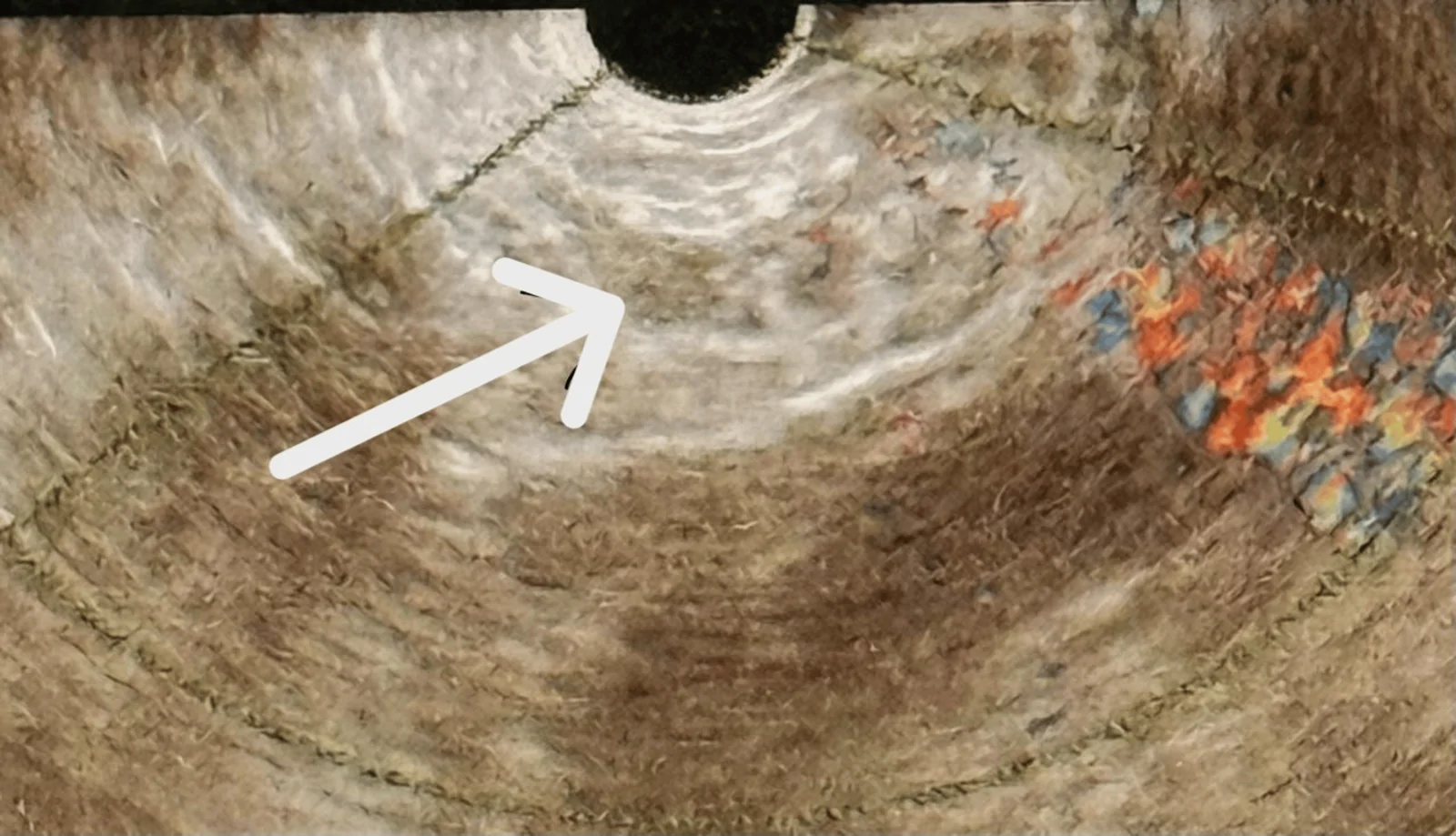
Endoscopic ultrasonography showing lobularity without honeycombing The arrow highlights the area of lobularity without honeycombing.

Additional investigations to rule out the extra-pancreatic causes of elevated pancreatic enzymes were carried out, all of which were unremarkable (IgG4, fecal calprotectin, anti-tissue transglutaminase antibodies, anti-nuclear antibody profile, angiotensin-converting enzyme level, immunoglobulin levels, protein electrophoresis) (Table 2). Next-generation sequencing for the pancreatitis panel was unremarkable. Pathogenic variants of PRSS1, SPINK1, CFTR, CASR, CPA1, CEL-HYB1, TRPV6, and CTRC genes were not detected. However, a variant of uncertain significance (CEL: c.1183C>T) was detected (gene: CEL (+) NM_001807.6; location: exon 9; variant: c.1183C>T (p.Pro395Ser); zygosity: heterozygous; inheritance: autosomal dominant).

**Table 2:** Additional laboratory investigations of the patient

Investigation	Patient values	Reference range
Immunoglobulin A	2 gm/L	0.7-4 gm/L
Immunoglobulin G	11.2 gm/L	5.4-15.8 gm/L
Immunoglobulin G4	0.59 gm/L	0.03-2 gm/L
Fecal calprotectin	<5 mcg/gm	<50 mcg/gm
Anti-tissue transglutaminase antibody immunoglobulin G	0.3 U/mL	<7 U/mL
Anti-tissue transglutaminase antibody immunoglobulin A	0.2 U/mL	<7 U/mL
Anti-nuclear antibody profile	Negative	Negative
Angiotensin-converting enzyme	24 U/L	12-68 U/L
Parathyroid hormone	59 pg/mL	15-68.3 pg/mL
Albumin	4.42 gm/dL	3.97-4.95 gm/dL
Alpha-1 globulin	0.23 gm/dL	0.21-0.35 gm/dL
Alpha-2 globulin	0.50 gm/dL	0.51-0.85 gm/dL
Beta-1 globulin	0.52 gm/dL	0.34-0.52 gm/dL
Beta-2 globulin	0.34 gm/dL	0.23-0.47 gm/dL
Gamma globulin	1.01 gm/dL	0.8-1.35 gm/dL

Due to the asymptomatic status and negative laboratory and imaging findings, the patient was put on close follow-up with serial measurement of pancreatic enzyme levels (Table 3) and a repeat EUS after two years (which revealed the same findings vide supra)(isolated lobularity without honeycombing has low specificity and can be seen in normal aging or fatty infiltration) (Figure 7). He was continued on his regular medications for hypertension, dyslipidemia, and type 2 diabetes. During this period, the patient remained asymptomatic. Both of his children underwent pancreatic enzyme estimation to determine any familial pattern of the syndrome, which were within the normal range.

**Table 3:** Serial pancreatic enzyme levels of the patient UNL: upper normal limit

Investigation	January 2024	January 2025	December 2025	April 2026
Serum amylase (UNL: 110 U/L)	236 U/L	286 U/L	431 U/L	520 U/L
Serum lipase (UNL: 60 U/L)	257 U/L	314 U/L	992 U/L	956 U/L

**Figure 7:**
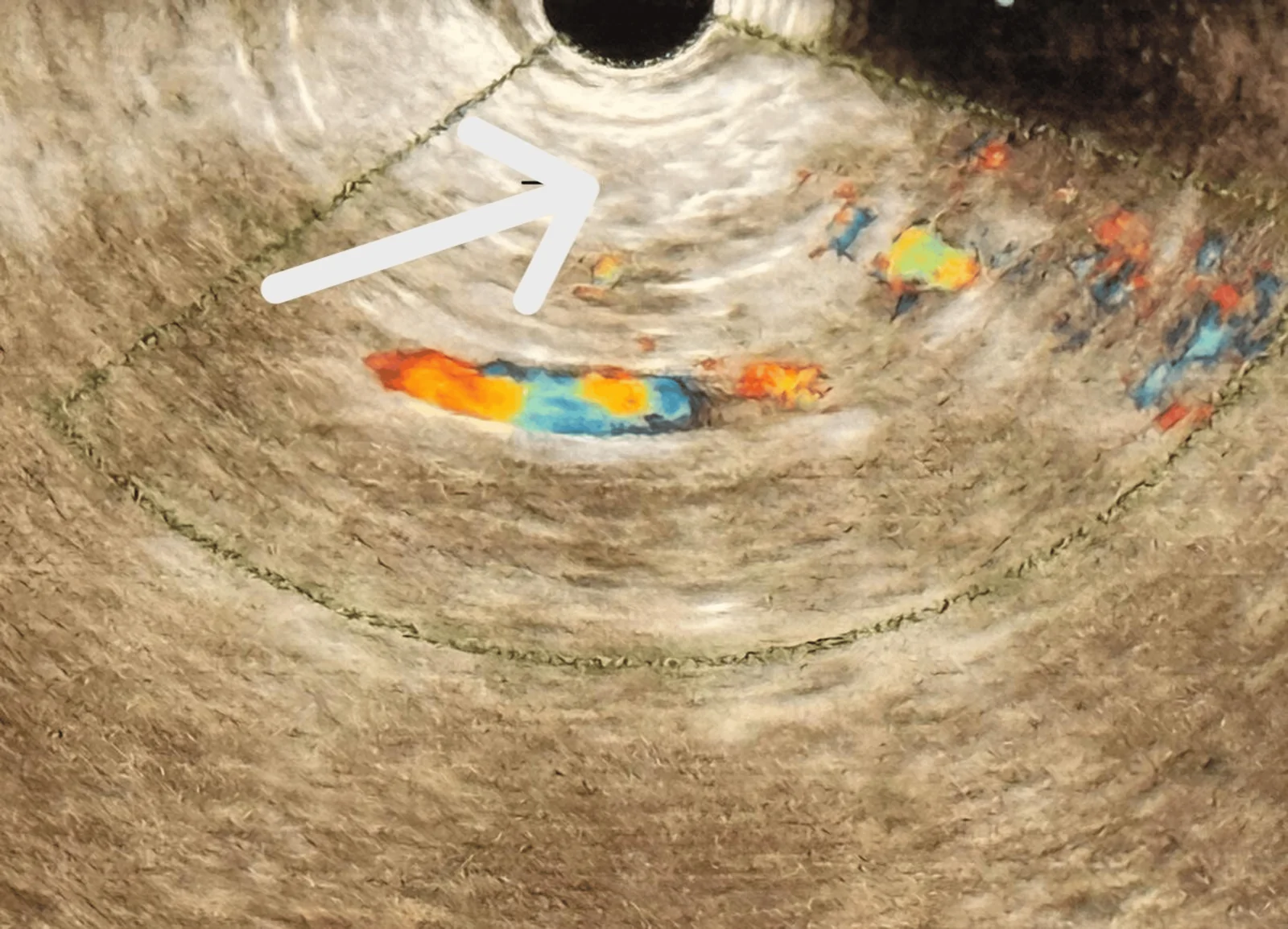
Repeat endoscopic ultrasonography showing lobularity without honeycombing The arrow highlights the area of lobularity without honeycombing.

A clinical diagnosis of benign pancreatic hyperenzymemia or Gullo's syndrome was made in view of persistent enzyme elevation for more than a year in the absence of clinical, laboratory, or radiological features.

## Discussion

Benign pancreatic hyperenzymemia or Gullo's syndrome is characterized by an increase in serum levels of exocrine pancreatic enzymes (amylase, isoamylase, lipase, and/or trypsin) in an otherwise healthy patient with an absence of clinical and imaging features of pancreatic pathology. It was first described in 1996 by the Italian gastroenterologist Lucio Gullo in a cohort of 18 adults displaying no symptoms [2]. Since then, only a few case reports have been published [3-9]. It is often an incidental finding and is commonly picked up during regular body check-ups or preoperative work-ups.

The syndrome shows incidence across all ages with greater documentation in the older age group. Its frequency is higher in men than women (1.5:1) [9]. It has been reported to occur sporadically as well as in familial forms [2]. Its prevalence and etiology are not known.

Multiple pancreatic enzymes are elevated in a majority of cases (as in our case), but cases with a single elevated enzyme have also been reported (5-10%) [11]. The syndrome persists over time, but enzyme levels are known to fluctuate transiently, with intervals during which levels are within the normal range. Day-to-day fluctuations in enzyme values have also been reported [9]. As in our case, serum lipase levels are usually more elevated than serum amylase levels [9].

In adults, an exhaustive investigation and close follow-up are necessary because pancreatic hyperenzymemia has been described as an early sign of pancreatic cancer [8]. Establishment of benign pancreatic hyperenzymemia requires at least one year of asymptomatic follow-up with normal imaging. In our patient, we have two years of asymptomatic follow-up with paired normal imaging.

For the diagnosis of early chronic pancreatitis, EUS is superior to ultrasonography, CT scan, or MRCP. According to JPS in 2019 [10], more than two parenchymal criteria on EUS or ductal abnormalities on endoscopic retrograde cholangiopancreatography (ERCP)/MRCP in the presence of one of the clinical criteria can be diagnostic of early chronic pancreatitis. In our case, pancreatic enzyme elevation was the only positive clinical criterion; there were no ductal abnormalities on EUS or MRCP; there was a single parenchymal criterion on EUS, namely, lobularity without honeycombing; and follow-up EUS after two years showed similar findings. Lobularity on EUS correlates with lobular fibrosis or fat deposition [10]. In studies looking at the natural history of early chronic pancreatitis, progression of EUS findings in 2-3 years is noted [10]. Thus, diagnosis of early chronic pancreatitis was ruled out in our case. While investigating pancreatic hyperenzymemia, a combination of EUS and MRCP is better than any single modality [12].

As the patient was on multiple drugs, drug-induced pancreatitis was considered (dipeptidyl peptidase 4 (DPP-4) inhibitors such as linagliptin are implicated), but was not the case in view of absent clinical and imaging features of pancreatitis and no changes in the pancreatic enzyme levels on discontinuation. Non-pancreatic causes of pancreatic enzyme elevation include multiple etiologies [1] which were satisfactorily ruled out in our case by clinical, imaging, and laboratory findings.

The quality of life of patients with benign pancreatic hyperenzymemia remains comparable to healthy individuals [13]. They only require reassurance regarding the benign, non-progressive nature of the syndrome and that no intervention is needed. This helps avoid repeated and expensive investigations and hospitalizations.

Multiple etiological mechanisms have been hypothesized but not proven. A sphincter of Oddi dysfunction, without becoming severe enough to cause symptomatology, was proposed by Frulloni et al. [14]. Cook et al. described a defect in an intracellular pathway (from the trans-Golgi network to the basolateral membrane through which newly synthesized enzymes reach the circulation) which leads to an increased enzyme release into circulation [15].

The role of genes commonly associated with pancreatic diseases such as PRSS1, SPINK1, and CFTR has been reliably ruled out [2,4,9,13]. A novel finding in our case was the presence of a variant of the CEL gene (CEL: c.1183C>T). This variant is of unknown clinical significance at present. Further testing in a larger population and in other patients with Gullo's syndrome may help determine its significance for the syndrome.

## Conclusions

In our case, asymptomatic presentation, persistent fluctuating elevation of pancreatic enzymes, and unremarkable imaging findings led to the diagnosis of Gullo's syndrome. It is rare and a diagnosis of exclusion at present. Persistent asymptomatic elevation of pancreatic enzymes should alert a clinician to the possibility of Gullo's syndrome. Normal imaging of the pancreas strengthens the possibility. An accurate and early diagnosis of Gullo's syndrome is beneficial for patients in terms of avoiding unnecessary investigations, unwarranted hospitalizations, and associated high costs. The mainstay of management remains reassurance of the patient regarding the benign nature of the disease. It is essential to emphasize that patients with this syndrome have a normal lifespan and a normal quality of life. The etiology and pathogenesis of Gullo's syndrome are not clear at present. As a familial type of Gullo's syndrome has been described, next-generation gene sequencing in patients and their families may lead to the identification of specific variants, which are unknown at present. 
